# Hemophagocytic Lymphohistiocytosis After Trauma Due to a Motor Vehicle Accident: A Case Report

**DOI:** 10.7759/cureus.20756

**Published:** 2021-12-27

**Authors:** Hanan S Tanbal, Hashem A Al-Dalooj, Ali H Al Qattan, Hassan E Al Abbas, Murtadha A Al Nas

**Affiliations:** 1 Hematology, Qatif Central Hospital, Qatif, SAU; 2 Medicine, Imam Abdulrahman Bin Faisal University, Dammam, SAU

**Keywords:** intravenous immunoglobulin (ivig), secondary hemophagocytic lymphohistiocytosis (hlh), hemophagocytic lymphohistiocytosis (hlh), darbepoetin, anemia, motor vehicle accident, trauma

## Abstract

Hemophagocytic lymphohistiocytosis (HLH) is an underdiagnosed, rare clinical syndrome, in particular secondary HLH, which mostly affects adults. HLH can be caused by malignancy, infections, autoimmune disorders, and, rarely, trauma. Here, we present the case of a patient who presented with anemia not responding to blood transfusion but improved after treatment with intravenous immunoglobulin. This case aims to highlight a rare presentation of this disease (HLH secondary to trauma) and to discuss the current HLH diagnostic criteria.

## Introduction

Hemophagocytic lymphohistiocytosis (HLH) is a relatively rare systemic inflammatory clinical syndrome that can be associated with various conditions, such as neoplastic, infectious, autoimmune, or hereditary diseases [1]. The primary form, familial HLH, typically presents early in life, is inherited as a recessive trait, and has been well-studied. The secondary form, adult-onset or acquired HLH, is often secondary to an underlying condition, such as infection, malignancy, or autoimmune disease [2].

HLH results when vital regulatory pathways responsible for the downregulation of immune responses are disturbed. This results in persistent activation of antigen-presenting cells and T lymphocytes, leading to a hyperinflammatory state [3].

HLH usually begins with acute or subacute, nonspecific, general symptoms that can vary depending on the presence of underlying disease, infectious triggers, and involvement of internal organs. The main features include enlarged lymphohematopoietic organs, continuous high fever (38.5°C), and cytopenias. Internal organ involvement is frequent and often leads to multiorgan failure. The spleen and the liver are the most frequently involved organs and can present as encephalopathy, ascites, veno-occlusive disease, or nontraumatic splenic rupture [4].

The most widely used criteria to diagnose HLH is based on a 2004 study’s inclusion criteria and were designed mainly for primary HLH in the pediatric population [5,6].

## Case presentation

A 22-year-old male with a known glucose-6-phosphate-dehydrogenase (G6PD) deficiency was admitted under the care of the orthopedic unit as a case of a pedestrian motor vehicle accident. Upon physical examination, the patient was vitally stable, conscious, alert, and oriented to time, place, and person. He appeared in pain but was not in respiratory distress. The lower limb examination revealed right femur shaft open fracture, left knee tenderness, and swelling suspicious for fracture with a left thigh open wound. The upper limb examination was unremarkable. The abdominal examination showed a soft and lax abdomen with no tenderness, no palpable spleen, or signs of hepatomegaly. Chest, cardiovascular, and neurological examinations were unremarkable.

The hematology unit was consulted due to the patient’s rapid drop in hemoglobin level from 10.0 g/dL to 7.3 g/dL with no obvious external source of bleeding, while an internal source of bleeding was ruled out by a pan CT. Upon further evaluation by the hematology team, the clinical history was negative for chest pain, shortness of breath, palpitations, or headache. Upon physical examination, the patient’s vitals were as follows: temperature of 37.2ºC, heart rate of 118 beats per minute, blood pressure of 106/55 mmHg, and oxygen saturation of 97% on room air. The physical examination revealed no changes from the patient’s baseline.

Table 1 summarizes the patient’s laboratory values upon admission, discharge, and follow-up after two months.

**Table 1:** Laboratory findings upon admission, discharge, and follow-up after two months. HCT: hematocrit; Hgb: hemoglobin; MCH: mean corpuscular hemoglobin; MCV: mean corpuscular volume; RBC: red blood cell; WBC: white blood cell

Laboratory finding	Upon admission	At discharge	Follow-up
Hgb	10.0 g/dL	8.9 g/dL	18.0 g/dL
WBC	22.89 × 10^3^/µL	11.53 × 10^3^/µL	3.59 × 10^3^/µL
RBC	3.61 × 10^6^/µL	3.4 × 10^6^/µL	7.1 × 10^6^/µL
Platelets	150 × 10^3^/µL	540 × 10^3^/µL	143 × 10^3^/µL
MCV	84.8 fL	85.6 fL	84.1 fL
MCH	27.7 PG	26.2 PG	25.5 PG
HCT	30%	29.1%	59.7%

Over the next seven days, the patient was given eight units of packed red blood cells (PRBCs) due to repeated drops of 1.5 g/dL or more in his hemoglobin levels within less than 24 hours of receiving the transfusions, with the lowest reading being 6.1 g/dL.

The patient was additionally transfused with two units of PRBCs to raise his hemoglobin to an acceptable level to perform the closed reduction of the right femur fracture with internal fixation and left intercondylar fracture fixation. The procedure went smoothly and there were no complications.

Postoperatively, the patient was vitally stable and afebrile. He was doing well, with the only complaint being mild pain at the surgical site. However, his hemoglobin levels dropped once again from 9.0 g/dL preoperatively to 7.9 g/dL over 24 hours and were not explained by intraoperative or postoperative bleeding. His liver function test revealed an alanine aminotransferase (ALT) of 166 U/L, serum aspartate aminotransferase (AST) of 192.2 U/L, total bilirubin of 56.7 g/L, and direct bilirubin of 28.3 g/L. A CT scan of the abdomen and pelvis obtained while evaluating the patient’s anemia did not demonstrate any internal bleeding or organomegaly. Urine analysis was negative for bilirubin and urobilinogen. The presence of reticulocytopenia of 0.6% suggested an impaired synthesis or release of red blood cells (RBCs) rather than hemolysis or hemorrhage as the cause of his anemia.

The patient was given a dose of darbepoetin, 80 mg subcutaneously, followed by 1 g/kg/day of intravenous immunoglobulin (IVIG) for the next two days to address the low hemoglobin level. Significant improvement in the patient’s hemoglobin (from 7.2 g/dL to 8.9 g/dL) and the clinical picture was seen three days after the administration of IVIG. The elevation in liver enzymes was most likely due to hepatic injury from preoperative cefazoline and the anesthetic agents that were used, which resolved spontaneously.

The patient’s rapid improvement after the administration of IVIG, the elevated serum ferritin of 625 µg/L, and lack of adequate response to blood transfusion support the diagnosis of HLH. Hemolysis secondary to G6PD deficiency was not considered because G6PD deficiency patients usually have ample response to blood transfusion. Additionally, there was no evidence of hemolysis in the patient’s laboratory investigations. A direct Coombs test was negative which excluded the diagnosis of autoimmune hemolytic anemia. Moreover, an indirect Coombs test was also negative which excluded RBC incompatibility. The patient was offered a bone marrow biopsy to confirm the diagnosis of HLH. However, it was not performed due to the patient’s condition and refusal of the procedure.

## Discussion

The diagnosis of HLH is a difficult one to make. Currently, the most widely accepted criteria for diagnosing both primary and secondary HLH is not based on well-validated data or clinical experience but rather on the inclusion criteria of a single clinical trial (NCT00426101) conducted in 2004. The study’s population consisted solely of pediatric patients with the majority having primary HLH. Therefore, these criteria were not designed to diagnose secondary HLH in adults [5].

While very few cases of HLH secondary to trauma have been reported, Erdoğan et al. reported the case of a six-year-old patient who suffered head trauma after an out-of-car traffic accident and developed secondary HLH with a presentation more closely matching the 2004 HLH criteria [7]. This can be attributed to the criteria’s original design [5].

Histopathological studies are crucial for diagnosing HLH. However, it was technically difficult to perform a bone marrow biopsy for our patient due to his multiple trauma sites. Therefore, the diagnosis was made based on the patient’s clinical background and his response to IVIG which supported our suspicion.

Trauma has been previously found to be a cause of T cell anergy. This T cell anergy has been found to contribute to unopposed monocyte activation because anergic T cells do not produce inhibitory interleukin (IL)-4, IL-10, or IL-13 [8]. This aberrant monocyte activation may be a contributing factor to the development of HLH in these patients.

Because secondary HLH is a challenging diagnosis to make, delaying the initiation of therapy until diagnostic criteria are met may affect the patient’s prognosis poorly [6,9]. One of the difficulties, such as in this case report, is obtaining a bone marrow biopsy; therefore, treatment should not be delayed while awaiting a trauma patient to be fit for a bone marrow biopsy.

While there is no clear guideline for treating secondary HLH in adults, the general goal of treatment is to suppress the hyperinflammatory process, typically with a combination of the following: corticosteroids, IVIG, or IL-1 receptor antagonist [10-12]. In our patient, due to the detrimental effects of corticosteroids on wound and bone healing, treatment consisted solely of IVIG, and an excellent response was seen [13,14].

## Conclusions

There is an extremely limited number of reported cases of secondary HLH post-trauma in adults. This article aims to build upon the available literature and to set forth the features of a possible presentation of post-traumatic secondary HLH in adults. Secondary HLH in adults is a rare disease with no reliable and validated clinical criteria for diagnosis. The gap in knowledge regarding secondary HLH in adults extends further into the management of the disease where only limited guidelines are available.
